# Supplementary material for: A Comparative Study of Binding Interactions between Proteins and Flavonoids in Angelica Keiskei: Stability, α-Glucosidase Inhibition and Interaction Mechanisms
Source: Int J Mol Sci. 2023 Apr 1;24(7):6582. doi: 10.3390/ijms24076582 (PMC10095106; doi:10.3390/ijms24076582)
Supplement: Supplementary file 1 [file ijms-24-06582-s001.zip › ijms-2173156-supplementary.pdf]

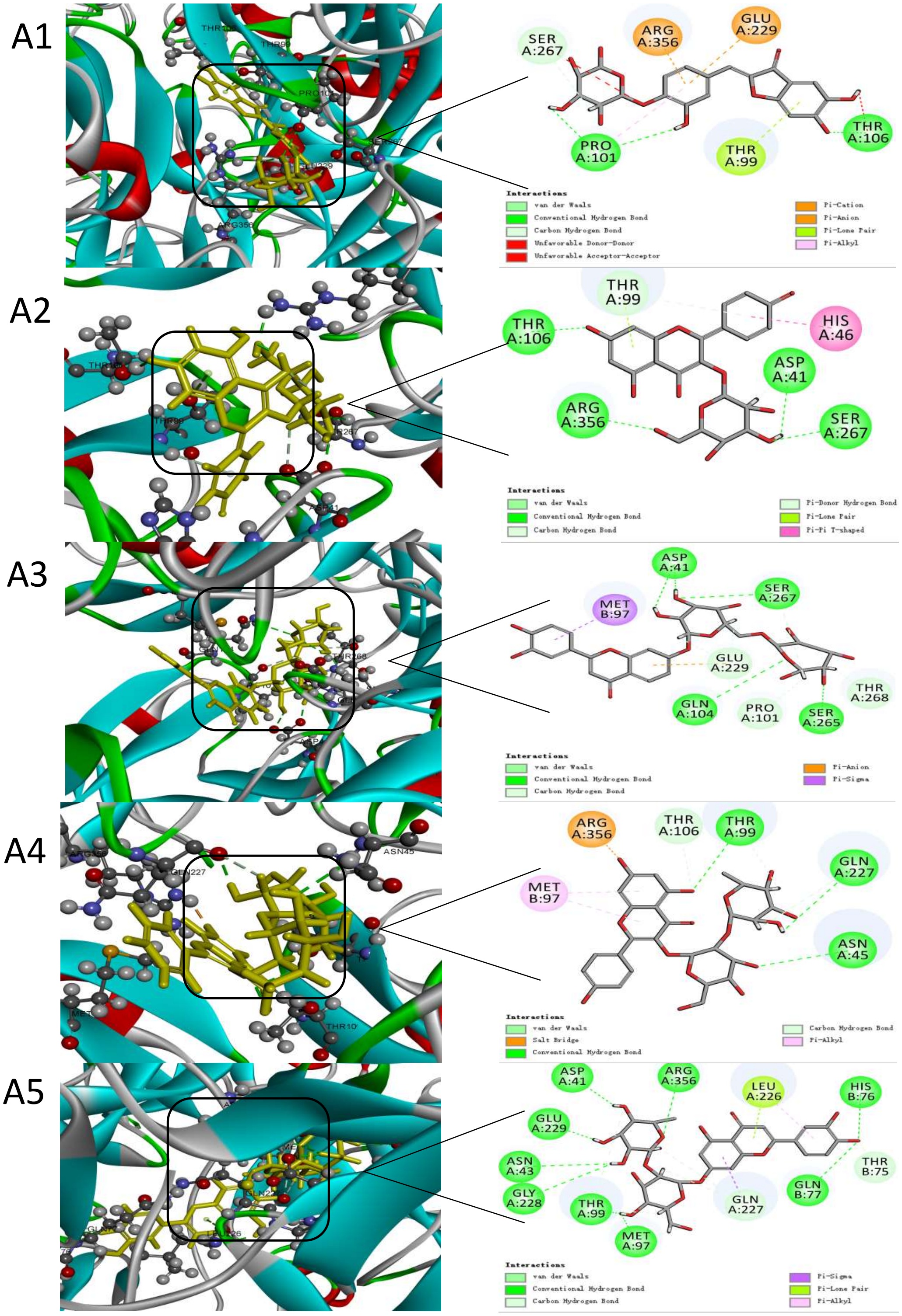

A1
SER A:267
ARG A:356
GLU A:229
PRO A:101
THR A:99
THR A:106
Interactions
van der Waals
Conventional Hydrogen Bond
Carbon Hydrogen Bond
Unfavorable Donor-Donor
Unfavorable Acceptor-Acceptor
Pi-Cation
Pi-Anion
Pi-Lone Pair
Pi-Alkyl
A2
THR A:99
THR A:106
HIS A:46
ASP A:41
ARG A:356
SER A:267
Interactions
van der Waals
Conventional Hydrogen Bond
Carbon Hydrogen Bond
Pi-Donor Hydrogen Bond
Pi-Lone Pair
Pi-Pi T-shaped
A3
ASP A:41
SER A:267
MET B:97
GLU A:229
THR A:268
GLN A:104
PRO A:101
SER A:265
Interactions
van der Waals
Conventional Hydrogen Bond
Carbon Hydrogen Bond
Pi-Anion
Pi-Sigma
A4
ARG A:356
THR A:106
THR A:99
GLN A:227
MET B:97
ASN A:45
Interactions
van der Waals
Salt Bridge
Conventional Hydrogen Bond
Carbon Hydrogen Bond
Pi-Alkyl
A5
ASP A:41
ARG A:356
LEU A:226
HIS B:76
GLU A:229
ASN A:43
THR B:75
GLY A:228
THR A:99
GLN A:227
GLN B:77
MET A:97
Interactions
van der Waals
Conventional Hydrogen Bond
Carbon Hydrogen Bond
Pi-Sigma
Pi-Lone Pair
Pi-Alkyl

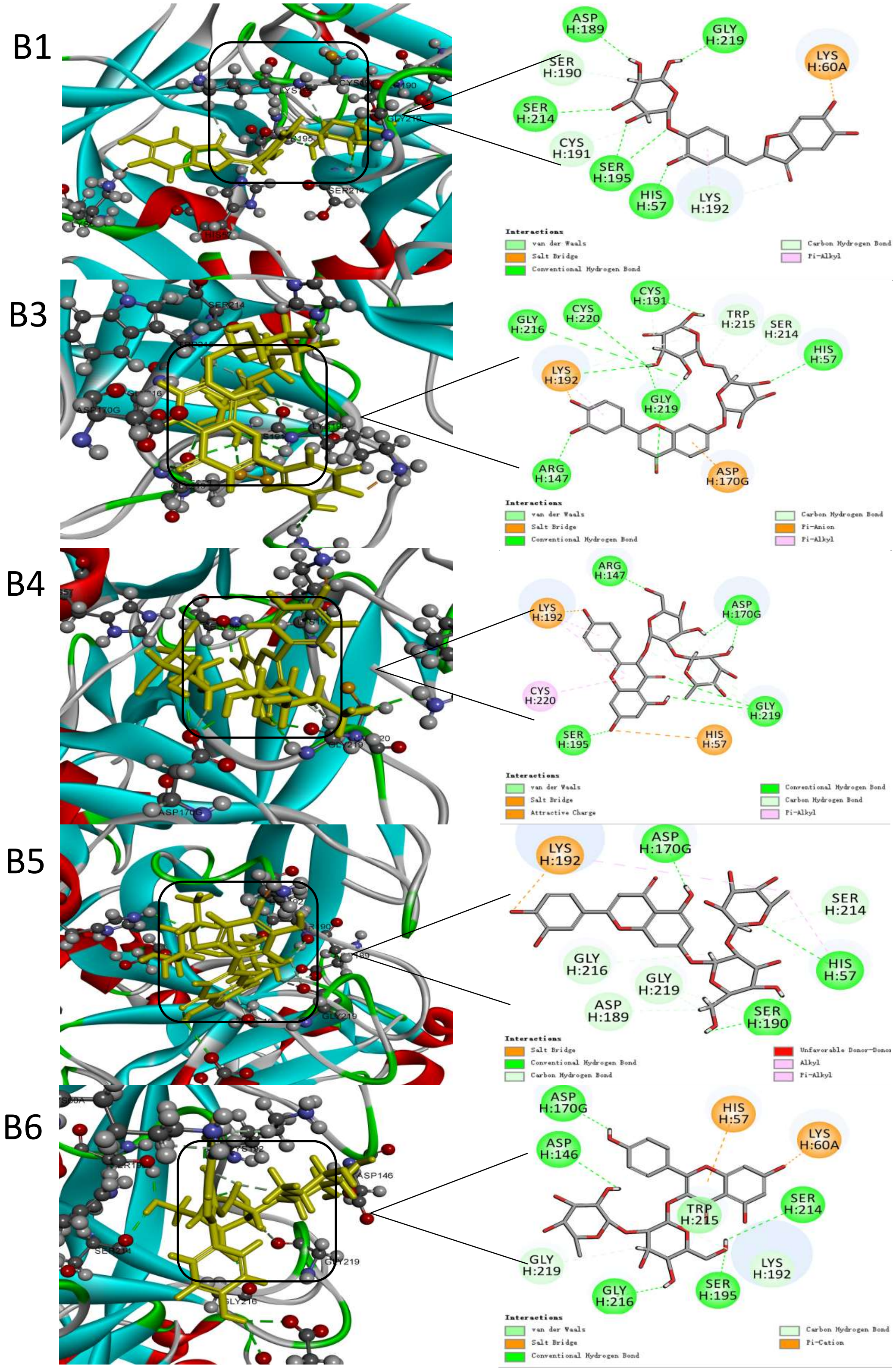
B1
ASP H:189
GLY H:219
SER H:190
LYS H:60A
SER H:214
CYS H:191
SER H:195
HIS H:57
LYS H:192
Interactions
van der Waals
Salt Bridge
Conventional Hydrogen Bond
Carbon Hydrogen Bond
Pi-Alkyl
B3
CYS H:191
CYS H:220
GLY H:216
TRP H:215
SER H:214
HIS H:57
LYS H:192
GLY H:219
ARG H:147
ASP H:170G
Interactions
van der Waals
Salt Bridge
Conventional Hydrogen Bond
Carbon Hydrogen Bond
Pi-Anion
Pi-Alkyl
B4
ARG H:147
LYS H:192
ASP H:170G
CYS H:220
GLY H:219
SER H:195
HIS H:57
Interactions
van der Waals
Salt Bridge
Attractive Charge
Conventional Hydrogen Bond
Carbon Hydrogen Bond
Pi-Alkyl
B5
LYS H:192
ASP H:170G
SER H:214
HIS H:57
GLY H:216
GLY H:219
ASP H:189
SER H:190
Interactions
Salt Bridge
Conventional Hydrogen Bond
Carbon Hydrogen Bond
Unfavorable Donor-Donor
Alkyl
Pi-Alkyl
B6
ASP H:170G
HIS H:57
LYS H:60A
ASP H:146
SER H:214
TRP H:215
GLY H:219
LYS H:192
GLY H:216
SER H:195
Interactions
van der Waals
Salt Bridge
Conventional Hydrogen Bond
Carbon Hydrogen Bond
Pi-Cation

C1

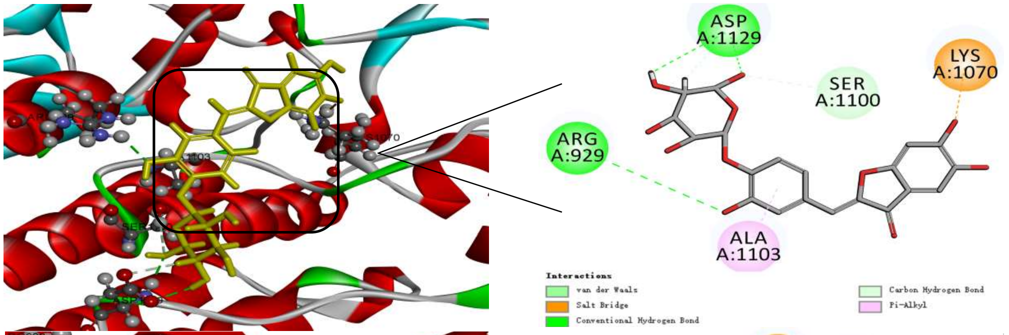
ASP A:1129
SER A:1100
LYS A:1070
ARG A:929
ALA A:1103
Interactions
van der Waals
Salt Bridge
Conventional Hydrogen Bond
Carbon Hydrogen Bond
Pi-Alkyl

C2

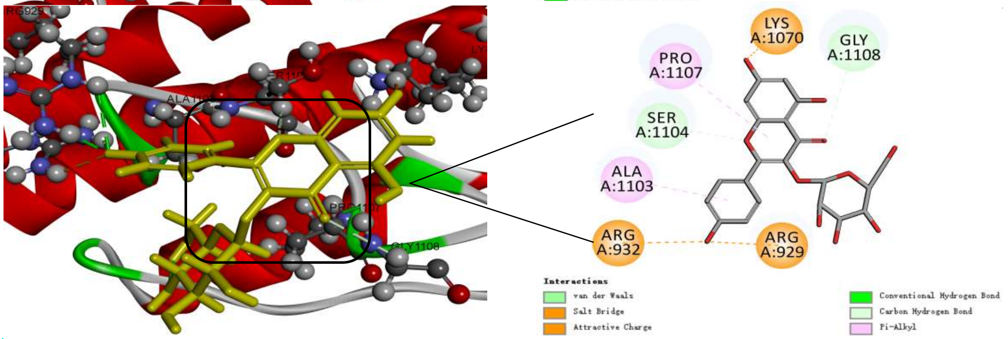
LYS A:1070
GLY A:1108
PRO A:1107
SER A:1104
ALA A:1103
ARG A:932
ARG A:929
Interactions
van der Waals
Salt Bridge
Attractive Charge
Conventional Hydrogen Bond
Carbon Hydrogen Bond
Pi-Alkyl

C3

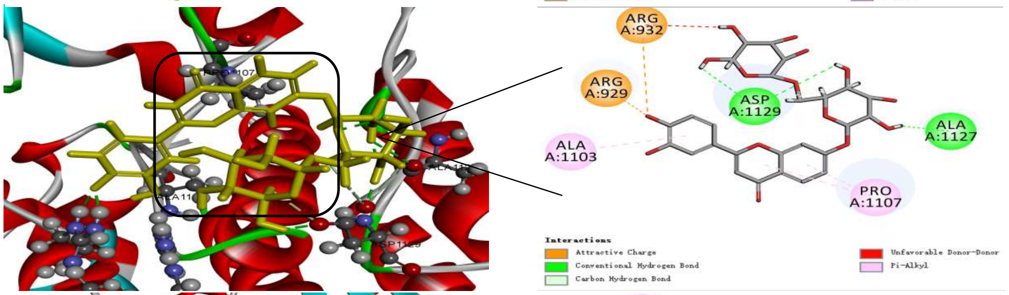
ARG A:932
ARG A:929
ASP A:1129
ALA A:1127
ALA A:1103
PRO A:1107
Interactions
Attractive Charge
Conventional Hydrogen Bond
Carbon Hydrogen Bond
Unfavorable Donor-Donor
Pi-Alkyl

C5

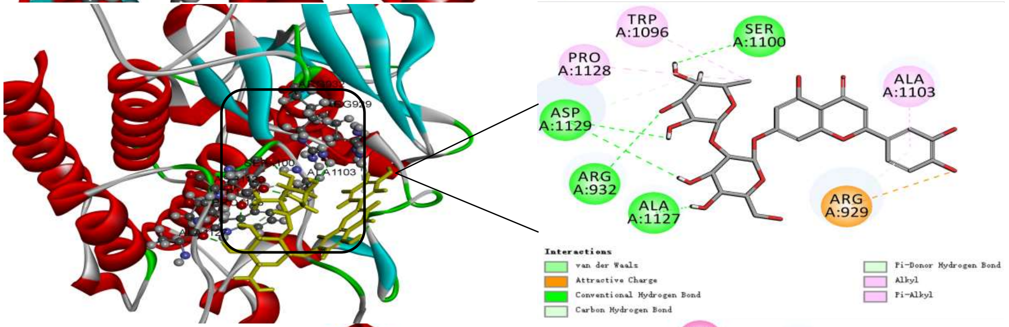
TRP A:1096
SER A:1100
PRO A:1128
ALA A:1103
ASP A:1129
ARG A:932
ALA A:1127
ARG A:929
Interactions
van der Waals
Attractive Charge
Conventional Hydrogen Bond
Carbon Hydrogen Bond
Pi-Donor Hydrogen Bond
Alkyl
Pi-Alkyl

C6

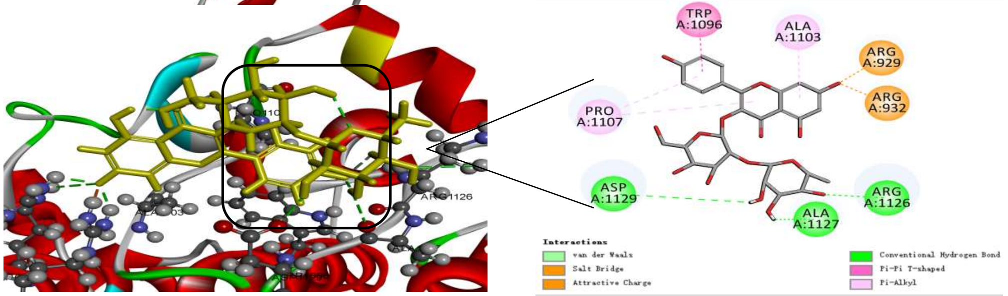
TRP A:1096
ALA A:1103
ARG A:929
PRO A:1107
ARG A:932
ASP A:1129
ARG A:1126
ALA A:1127
Interactions
van der Waals
Salt Bridge
Attractive Charge
Conventional Hydrogen Bond
Pi-Pi T-shaped
Pi-Alkyl

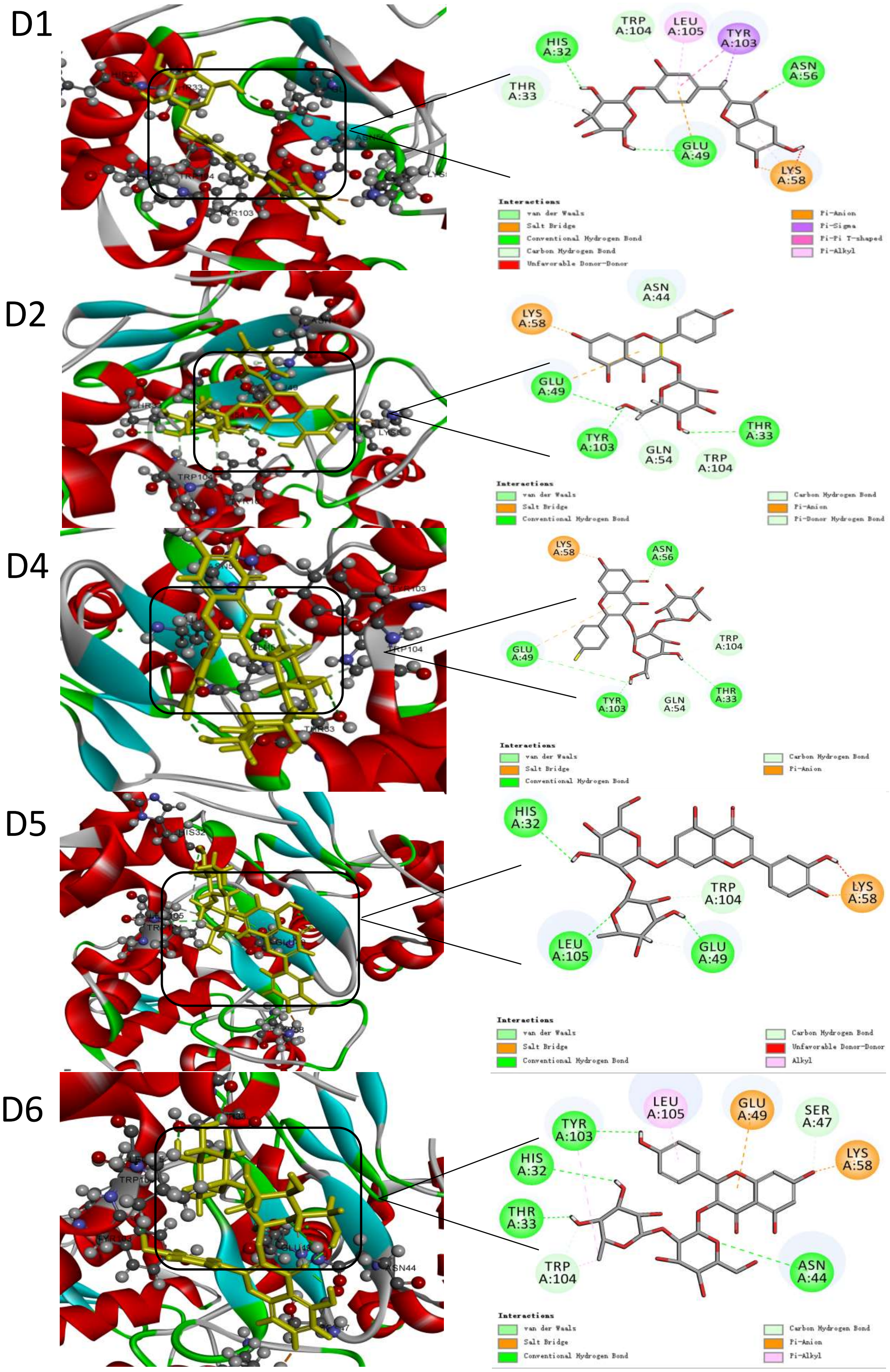

D1
HIS A:32
THR A:33
TRP A:104
LEU A:105
TYR A:103
ASN A:56
GLU A:49
LYS A:58
Interactions
van der Waals
Salt Bridge
Conventional Hydrogen Bond
Carbon Hydrogen Bond
Unfavorable Donor-Donor
Pi-Anion
Pi-Sigma
Pi-Pi T-shaped
Pi-Alkyl
D2
ASN A:44
LYS A:58
GLU A:49
TYR A:103
GLN A:54
TRP A:104
THR A:33
Interactions
van der Waals
Salt Bridge
Conventional Hydrogen Bond
Carbon Hydrogen Bond
Pi-Anion
Pi-Donor Hydrogen Bond
D4
LYS A:58
ASN A:56
GLU A:49
TRP A:104
TYR A:103
GLN A:54
THR A:33
Interactions
van der Waals
Salt Bridge
Conventional Hydrogen Bond
Carbon Hydrogen Bond
Pi-Anion
D5
HIS A:32
TRP A:104
LYS A:58
LEU A:105
GLU A:49
Interactions
van der Waals
Salt Bridge
Conventional Hydrogen Bond
Carbon Hydrogen Bond
Unfavorable Donor-Donor
Alkyl
D6
LEU A:105
GLU A:49
SER A:47
TYR A:103
LYS A:58
HIS A:32
THR A:33
TRP A:104
ASN A:44
Interactions
van der Waals
Salt Bridge
Conventional Hydrogen Bond
Carbon Hydrogen Bond
Pi-Anion
Pi-Alkyl

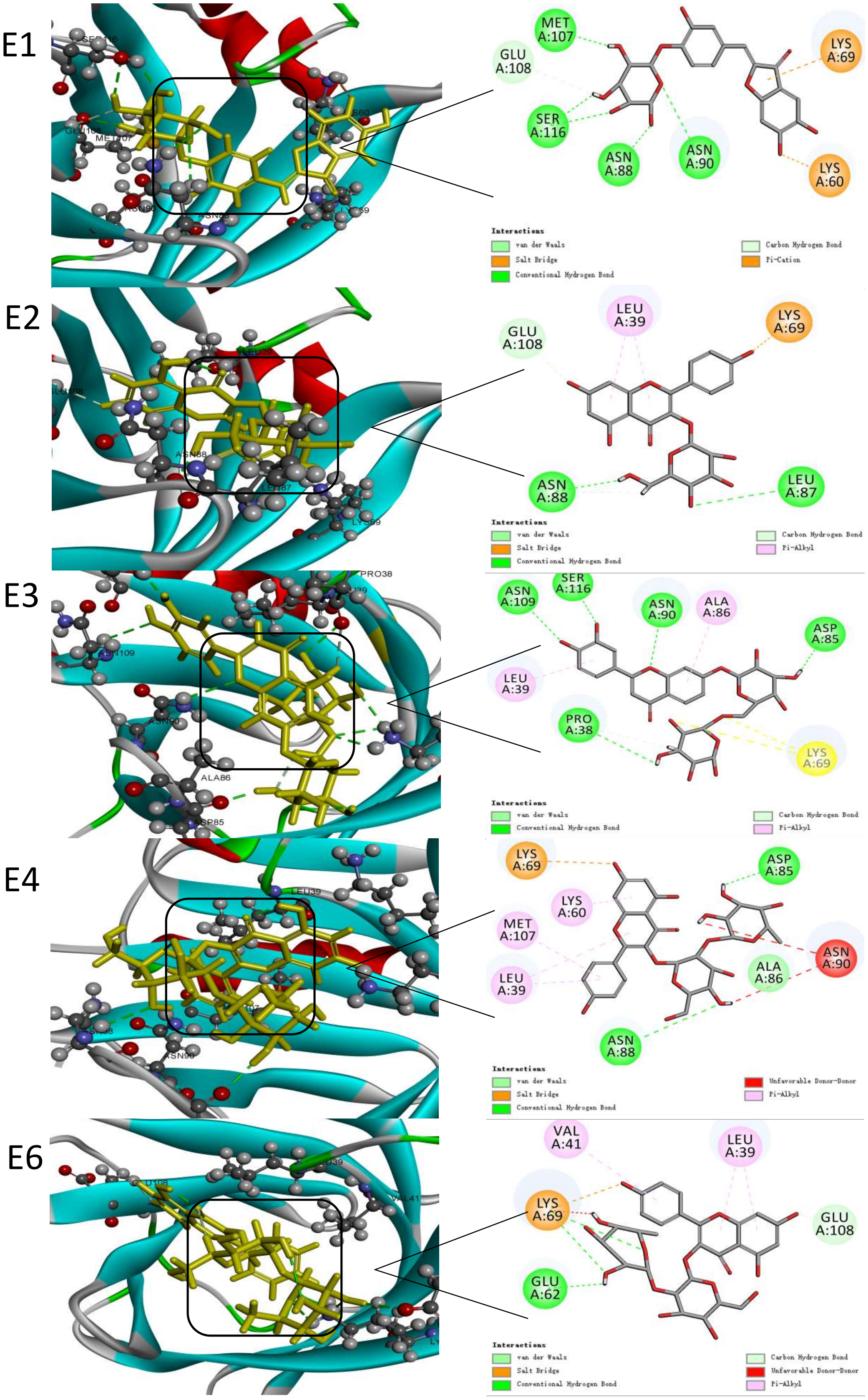
E1
MET A:107
GLU A:108
SER A:116
ASN A:88
ASN A:90
LYS A:69
LYS A:60
Interactions
van der Waals
Salt Bridge
Conventional Hydrogen Bond
Carbon Hydrogen Bond
Pi-Cation
E2
GLU A:108
LEU A:39
LYS A:69
ASN A:88
LEU A:87
Interactions
van der Waals
Salt Bridge
Conventional Hydrogen Bond
Carbon Hydrogen Bond
Pi-Alkyl
E3
PRO38
ASN A:109
SER A:116
ASN A:90
ALA A:86
ASP A:85
LEU A:39
PRO A:38
LYS A:69
ALA86
ASP85
Interactions
van der Waals
Conventional Hydrogen Bond
Carbon Hydrogen Bond
Pi-Alkyl
E4
LYS A:69
ASP A:85
LYS A:60
MET A:107
LEU A:39
ALA A:86
ASN A:90
ASN A:88
Interactions
van der Waals
Salt Bridge
Conventional Hydrogen Bond
Unfavorable Donor-Donor
Pi-Alkyl
E6
VAL A:41
LEU A:39
LYS A:69
GLU A:108
GLU A:62
Interactions
van der Waals
Salt Bridge
Conventional Hydrogen Bond
Carbon Hydrogen Bond
Unfavorable Donor-Donor
Pi-Alkyl

Figure S1. The docking diagrams of the flavonoids(1,2,3,4,5)/SPI(7S) (A), flavonoids(1,3,4,5,6) / SPI(7S) (B), flavonoids(1,2,3,5,6)/BSA (C), flavonoids(1,2,4,5,6)/α-La (D) and flavonoids(1,2,3,4,6) /β-Lg (E) complexes.
